# Insulin Induces Phosphorylation of Serine Residues of Translationally Controlled Tumor Protein in 293T Cells

**DOI:** 10.3390/ijms16047565

**Published:** 2015-04-03

**Authors:** Jeehye Maeng, Miyoung Kim, Hyukjin Lee, Kyunglim Lee

**Affiliations:** Graduate School of Pharmaceutical Sciences, College of Pharmacy, Ewha Womans University, Seoul 120-750, Korea; E-Mails: mengjeehye@hanmail.net (J.M.); ammykim@hanmail.net (M.K.); hyukjin@ewha.ac.kr (H.L.)

**Keywords:** insulin, Na,K-ATPase, phosphorylation, serine, TCTP

## Abstract

Insulin induces the activation of Na,K-ATPase while translationally controlled tumor protein (TCTP) inhibits this enzyme and the associated pump activity. Because binding of insulin with its membrane receptor is known to mediate the phosphorylation of multiple intracellular proteins, phosphorylation of TCTP by insulin might be related to the sodium pump regulation. We therefore examined whether insulin induces TCTP phosphorylation in embryonic kidney 293T cells. Using immunoprecipitation and Western blotting, we found that insulin phosphorylates serine (Ser) residues of TCTP. Following fractionation of the insulin-treated cells into cytosol and membrane fractions, phosphorylated TCTP at its Ser residue (p-Ser-TCTP) was detected exclusively in the cytosolic part and not in the membrane fraction. Phosphorylation of TCTP reached maximum in about 10 min after insulin treatment in 293T cells. In studies of cell-type specificity of insulin-mediated phosphorylation of TCTP, insulin did not phosphorylate TCTP in HeLa cells. Computational prediction and immunoprecipitation using several constructs having Ser to Ala mutation at potential p-Ser sites of TCTP revealed that insulin phosphorylated the serine-9 and -15 residues of TCTP. Elucidations of how insulin-mediated TCTP phosphorylation promotes Na,K-ATPase activation, may offer potential therapeutic approaches to diseases associated with vascular activity and sodium pump dysregulation.

## 1. Introduction

Insulin is known to activate Na,K-ATPase [1] and causes vasodilation via sodium pump activation [2]. In diabetic conditions, Na,K-ATPase function is impaired resulting in cardiac dysfunction [3]. Investigations on how insulin stimulates the sodium pump may help to understand the pathophysiology of diabetes and diabetes-induced hypertension. Mounting evidence indicates that insulin promotes the synthesis or phosphorylation of sodium pump, its translocation to the plasma membrane, and increases the intracellular concentrations of K^+^ [1,4,5,6,7,8,9,10]. It has been proposed that insulin-induced sodium pump regulation occurs via a still unknown mechanism, involving an as yet unknown cytoplasmic repressor [11].

We previously reported that translationally controlled tumor protein (TCTP), a house keeping protein implicated in various intracellular processes, such as apoptosis, cell cycle regulation, malignant transformations, and extracellular, cytokine-like activities [12], regulates Na,K-ATPase [13]. TCTP inhibits the pump activity of Na,K-ATPase by binding to third cytoplasmic domain of the enzyme’s α subunit [13], resulting in the development of systemic hypertension in TCTP-overexpressing transgenic mice (TCTP-TG) [14]. Inhibition of the Na,K-ATPase activity by TCTP overexpression is responsible for the hypercontractile reactivity of TCTP-TG mice [14].

It has been shown that insulin activates both α1 and α2 isoforms of Na,K-ATPase in rat adipocytes [11]. This effect of insulin was abolished in ghosts and plasma membranes isolated from adipocytes, implying the involvement of a diffusible inhibitor of Na,K-ATPase that is under regulation by insulin [11]. It was speculated that a labile regulatory molecule inhibits Na,K-ATPase and that insulin treatment releases this factor from the pump, causing the stimulation of Na,K-ATPase [11]. This suggests that both TCTP phosphorylation and its binding to Na,K-ATPase may be under the regulation of insulin. In this regard, an unknown kinase that phosphorylates TCTP and regulates the binding of TCTP with sodium pump was proposed [13]. We hypothesize that TCTP, a cytoplasmic repressor for Na,K-ATPase, may be a diffusible regulator for sodium pump and that insulin modifies TCTP causing it to be dissociated from Na,K-ATPase, thereby activating the pump. Because binding of insulin with its receptor [2] on the membrane, results in the phosphorylation of multiple intracellular proteins [15], we decided to investigate whether TCTP is one of the proteins phosphrorylated by insulin in human embryonic kidney 293T cells.

## 2. Results

### 2.1. Insulin Induces Phosphorylation of TCTP at Serine Residues and Promotes Its Translocalization to the Cytosol

**Figure 1:**
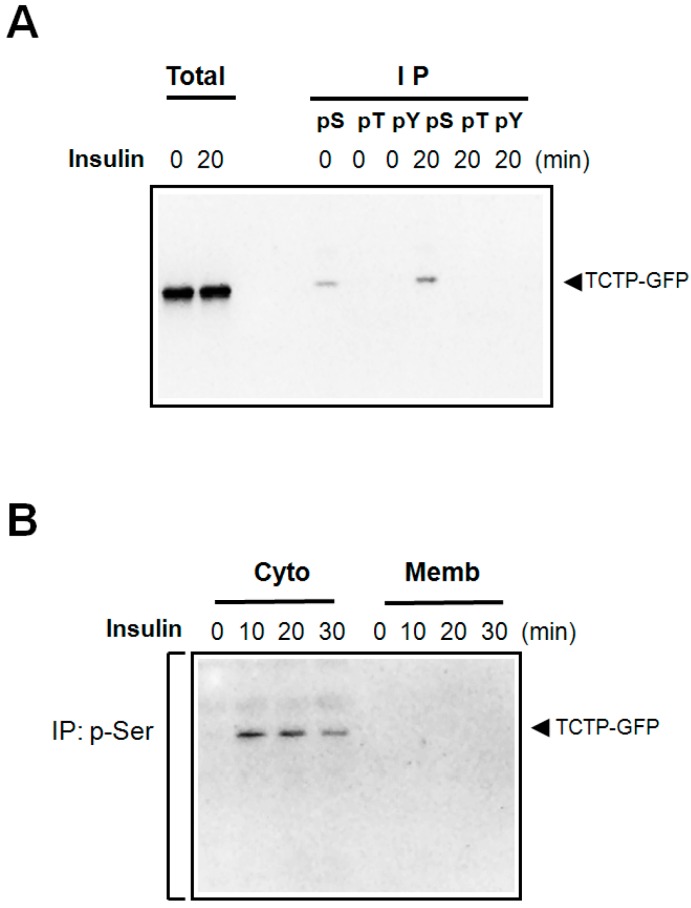
Insulin-induced phosphorylation of TCTP in 293T cells and the cytosolic localization of phosphorylated TCTP. (**A**) Following starvation, pEGFP-N1-TCTP-transfected 293T cells were treated with insulin at 100 nM concentrations for indicated times. Cells were harvested, and subsequently lysed in the lysis buffer containing detergent (1% Triton X-100) on ice. After brief sonication, samples were centrifuged at a 10,000 rpm for 10 min. Resulting supernatants were used as total cell lysates. For immunoprecipitation, anti-p-Ser-, -p-Tyr-, and -p-Thr-specific antibodies were used to precipitate the phosphorylated TCTP in cell lysates. The immune complexes so obtained were resolved in SDS-PAGE and then immunoblotted using anti-GFP-antibody; (**B**) Following transfection of pEGFP-N1-TCTP construct, 293T cells were treated with 100 nM insulin. Cell lysates thus obtained were fractionated into membrane and cytosolic fractions, as indicated in the Materials and Methods sections. Each fraction was then immunoprecipitated using anti-p-Ser antibodies, followed by immunoblotting with anti-GFP antibody.

Insulin is known to activate a number of kinases that activate signaling pathways. TCTP contains potential phosphorylation sites at its serine (Ser, S), threonine (Thr, T), and tyrosine (Tyr, Y) residues. To test whether insulin promotes phosphorylation of TCTP, lysates of 293T cells transfected with pEGFP N1-TCTP-GFP construct, were immunoprecipitated with anti-phospho-Ser-, -phospho-Thr-, and -phospho-Tyr-specific antibodies. Phosphorylated TCTP was detected through immunoblotting with anti-GFP antibody. As shown in Figure 1A, insulin treatment resulted in the phosphorylation of TCTP at its Ser residue(s), but not at Tyr and Thr residues.

Phosphorylation of proteins induces intracellular translocation of specific proteins, between membrane and cytosol. We examined whether insulin treatment affects the localization of TCTP by specifically detecting the p-Ser-TCTP in cytosol and plasma membrane of the transfected 293T cells treated with insulin. We obtained the membrane and cytosolic fractions of the insulin treated cells; obtained immunoprecipitates with anti-p-Ser-antibodies and subjected them to immunoblotting to detect p-Ser-TCTP using anti-GFP antibodies. Figure 1B shows that p-Ser-TCTP appears exclusively in the cytosolic and not in the membrane fractions. The amount of p-Ser-TCTP in the cytosol decreased at 30 min (Figure 1B), suggesting possible dephosphorylation by endogenous phosphatases.

### 2.2. Insulin Induces the Phosphorylation of both Endogenous and Exogenous TCTP in 293T Cells

**Figure 2:**
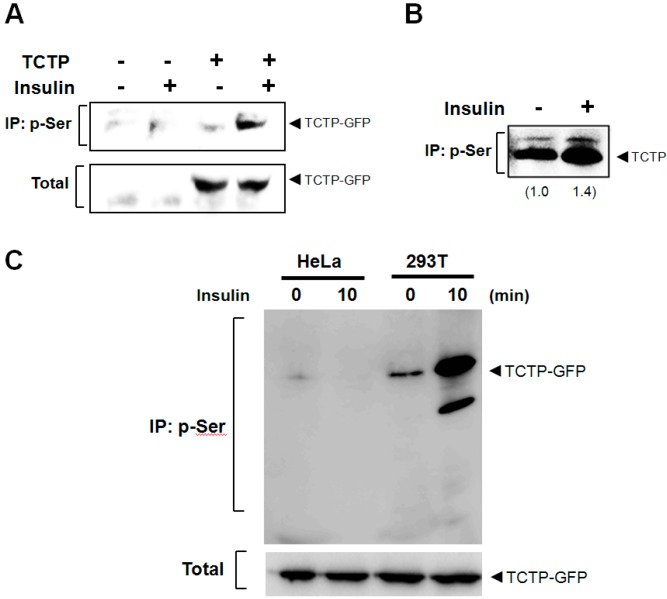
Insulin-induces phosphorylation of both endogenous and exogenous TCTP. (**A**) After transfection, the 293T cells to overexpress pEGFP-N1-TCTP construct, insulin was treated at a concentration of 100 nM. Following cytosolic preparation, TCTP phosphorylation at Ser residue(s) was examined through immunoprecipitation using anti-p-Ser antibody, followed by immunoblotting using anti-GFP antibody; (**B**) Endogenous TCTP phosphorylation at Ser residue following 100 nM insulin treatments in cytosolic fraction was determined by immunoprecipitation with anti-p-Ser antibody and Western blotting with anti-TCTP-specific antibody. Relative band intensities are calculated by Image J software (National Institute of Health, Bethesda, MD, USA) and are expressed as a fold increase of untreated cells; (**C**) Cells were transfected with pEGFP-N1-TCTP construct and were treated with 100 nM insulin for 10 min in HeLa and 293T cells, respectively. After cytosolic preparation, phosphorylated TCTP was detected by immunoprecipitation using anti-p-Ser antibodies and immunoblotting with anti-GFP antibody.

We examined whether the phosphorylation of TCTP by insulin depends on the origin of TCTP (*i.e.*, exogenous *vs.* endogenous origins) and the cell type (293T or others, such as HeLa). Exogenous TCTP was introduced by transfection. Cells were incubated with insulin and the cytosolic fraction was subjected to immunoprecipitation using anti-p-Ser-specific antibodies; and immunoblotting performed with anti-GFP or -TCTP-specific antibodies. We found that both exogenous (Figure 2A) and endogenous TCTPs (Figure 2B) are phosphorylated by insulin at Ser residues. We then tested whether insulin-promoted TCTP phosphorylation occurs also in cells other than 293T cells, such as human cervical adenocarcinoma HeLa cells. We found insulin-induced TCTP phosphorylation occurred only in 293T cells and not in HeLa cells (Figure 2C). This suggests that Ser phosphorylation of TCTP by insulin is a cell-type-specific phenomenon.

### 2.3. Insulin Phosphorylates TCTP at Ser-9 and -15 Residues

TCTP contains 8 Ser residues located at positions 9, 15, 37, 46, 53, 64, 82, and 98 in its primary structure. We attempted *in silico* prediction, using several servers that permit prediction of phosphoresidues, such as NetPhos, and PHOSIDA in rat TCTP, to determine which of the Ser residues of TCTP are phosphorylated by insulin. PHOSIDA [16], a phosphosite predictor, suggested phosphorylations at Ser-15, -37, -46, -53, -64, and -98 (data not shown) and NetPhos 2.0 that uses an artificial network [17] identified Ser residues at 9, 37 and 53 as potential phosphorylation sites (data not shown). Seven Ser residues of rat TCTP including Ser-9, -15, -37, -46, -53, -64, and -98 seem to be the candidate Ser sites phosphorylated by insulin.

**Figure 3:**
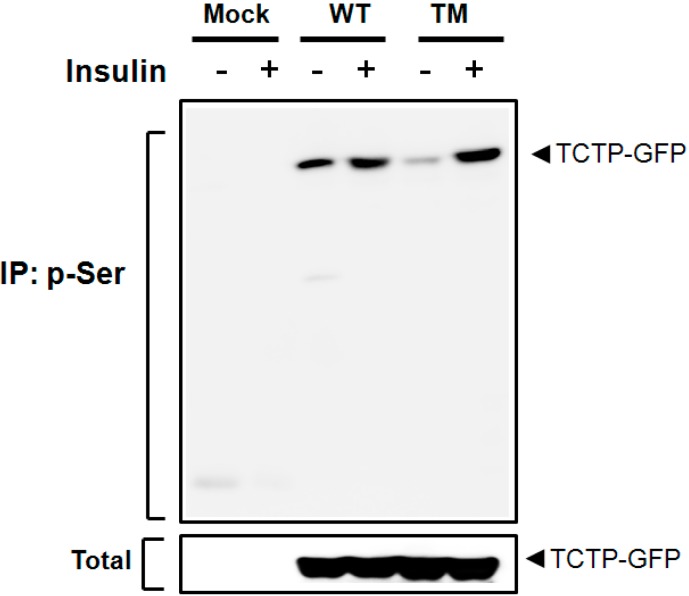
TCTP phosphorylation by insulin does not occur at Ser-46, -64, and -98 residues. Following transfection of 293T cells with pEGFP-N1-TCTP (WT) or pEGFP-N1-TCTPS46AS64AS98A (triple mutant, TM), cells were incubated with insulin-containing media. In order to determine whether Ser-46, -64, and -98 residues are involved in the phosphorylation sites for TCTP, alteration of p-Ser-TCTP in cytosolic fractions was assessed and compared in the presence or absence of Ser to Ala mutation in TCTP sequence. Following immunoprecipitation with anti-p-Ser antibody, immune complex was resolved and immunoblotted using anti-GFP antibody.

A biochemical study by Yarm identified Ser-46 and Ser-64 as phosphoresidues of TCTP [18]. Our own studies (unpublished) indicated that Ser-98 is a plausible site phosphorylated by Protein kinase C (PKC). In order to decide which of these residues are involved in insulin-induced TCTP phosphorylation, we generated constructs containing Ser to Ala point mutations at 46, 64, and 98. After overexpressing the wild-type TCTP (WT) or Ser to Ala point mutants (pEGFP-N1-TCTPS46AS64AS98A, TM) in 293T cells, the cells were treated with insulin to induce the TCTP phosphorylation. If those sites are involved in the insulin-induced TCTP phosphorylation, one would expect that p-Ser-TCTP in triple mutant cells would exhibit reduced level of phosphorylation compared to that of WT-TCTP-transfected cells. As shown in Figure 3, insulin treatment did not decrease the p-Ser-TCTP in triple mutant cells (TM), compared to that of WT-TCTP-transfected cells. Thus Ser-46, -64, and -98 residues of TCTP seem not involved in insulin-induced phosphorylation (Figure 3), leaving Ser-9, -15, -37, and -53 as the likely phosphorylation residues by insulin.

**Figure 4:**
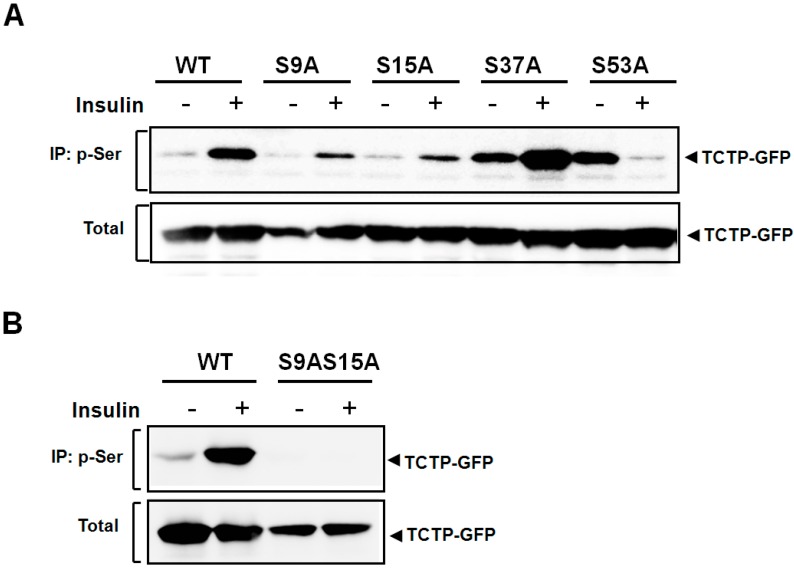
TCTP phosphorylation by insulin occurs only at Ser-9 and -15 residues. (**A**) To locate the potential phosphorylation residues of TCTP when insulin stimulates its phosphorylation, several Ser to Ala point mutants were designed and constructed. 293T cells were transfected with pEGFP-N1-WT, -S9A, -S15A, -S37A, and -S53A, serum-starved, and incubated with 100 nM insulin. After preparation of cytosolic fractions, it was precipitated with anti-p-Ser-specific antibody and immunoblotted using anti-GFP-specific antibody; (**B**) Constructs, pEGFP-N1-WT (WT) and double point mutant, pEGFP-N1-S9AS15A (S9AS15A) were transiently overexpressed in 293T cells by transfection, respectively. After serum starvation, cells were subjected to treatment with 100 nM insulin. Cytosolic fractions obtained were analyzed for p-Ser-TCTP by immunoprecipitation and immunoblotting.

To confirm the sites in TCTP phosphorylated by insulin, we generated constructs bearing Ser to Ala mutations at the Ser-9, -15, -37, and -53 of TCTP (pEGFP-N1-S9A, -S15A, -S37A, and -S53A), transfected 293T cells with them and treated the transfected cells with insulin to induce TCTP phosphorylation. This study showed that both S9A and S15A mutant constructs exhibited reduced phosphorylation by insulin compared to that of wild-type TCTP (WT) (Figure 4A). Insulin-induced phosphorylation was attenuated in the S9A and S15A mutants and the reduction was similar in the two cases. Therefore, it appears that Ser-9 and -15 residues are the sites independently involved in TCTP phosphorylation to a comparable degree. In contrast, Ser-37 and -53 residues appear not be the sites for insulin-induced TCTP phosphorylation since both S37A- and S53A-transfected cells had increased basal levels of p-Ser-TCTP.

We also confirmed that Ser-9 and -15 are the sites of insulin-promoted TCTP phosphorylation in studies on the phosphorylation of S9A and S15A double mutant (pEGFP-N1-S9AS15A). In cells overexpressing S9AS15A TCTP, there was a total absence of phosphorylation of TCTP by insulin (Figure 4B). This confirms our conclusion that both Ser-9 and -15 residues serve as major phosphosites for insulin-induced phosphorylation in 293T cells.

## 3. Discussion

TCTP has been shown to be phosphorylated by polo-like kinase (Plk) at its Ser-46 and -64 residues in a hierarchical manner [18]. The present study demonstrates that Ser-9 and -15 of TCTP are the sites for phosphorylation by insulin. Insulin-mediated phosphorylation of TCTP at Ser-9 does not appear to require phosphorylation at Ser-15 as a prerequisite. This is supported by our observation that the degree of insulin-promoted phosphorylation of TCTP and reduction of p-Ser-TCTP was the same in cells expressing S9A and S15A mutants (Figure 4A). Additionally, we noted that insulin-induced phosphorylation sites, Ser-9 and -15, and its flanking sequences showed high degree of conservation among humans, rodents and chickens (data not shown), suggesting commonality in signalings through phylogeny. The basal phosphorylation and differential regulation by insulin in S37A and S53A mutants (Figure 4A) need further clarification.

It has been suggested that an unknown cytoplasmic factor regulated by insulin, may regulate the pump activity of Na,K-ATPase [11]. The present study demonstrated that insulin induces phosphorylation of cytosolic TCTP at its Ser residue(s). The observed specific localization of the phosphorylated TCTP in the cytosol (Figure 1B) implies that phosphorylation of TCTP by insulin may induce the translocation of TCTP from plasma membrane to cytosol, and also that TCTP may be a hitherto unidentified diffusible regulator of Na,K-ATPase. Our hypothesis proposes that TCTP itself may be regulated by insulin to activate the sodium pump by dissociating it from membrane sodium pump. To confirm the insulin-induced translocation of TCTP, it is necessary to track movement of TCTP between the cytosol and membrane fractions by techniques, such as immunofluorescence, which can help visualize translocation events. We propose the hypothetical mechanism that involves serial events including insulin promoted subcellular localization of TCTP, escape of Na,K-ATPase from TCTP suppression, and the stimulation of pump activity. Investigation on this hypothetical mode of insulin-induced modification of TCTP and sodium pump activation is underway in our researches. Elucidations of how insulin activates the sodium pump may help to understand the mechanism underlying insulin-induced vascular relaxation, in the context of TCTP phosphorylation.

Insulin-induced TCTP phosphorylation appears to be cell-type-specific because in our hands insulin did not induce TCTP phosphorylation in HeLa cells as it did in 293T cells (Figure 2C). Previously, it was speculated that insulin receptor is non-functional in spite of its endogenous expression in HeLa cells [19], indicating that insulin-induced signaling can vary among different types of cells. This may explain the disparity in the localization of signaling molecules and reactivity of insulin receptor [20] and in the tissue-specific regulation and signalings of sodium pump by insulin [1,4,5,6,7,8,9,10].

Regulation of TCTP can occur at transcriptional, translational, and/or post-translational levels. It is well established that TCTP functionally responds to various physiological and pathological stimuli at transcriptional and translational levels [12,21]. But the posttranslational regulation of TCTP is largely unknown, except that TCTP is phosphorylated by Plk [18]. Yarm suggested that Ser-46 and -64 residues are the sites for phosphorylation by mitotic polo-like kinase (Plk) during mitotic spindle regulation in mitosis [18]. Protein phosphorylation is a highly controlled post-translational modification and is a mechanism regulating protein function, localization, stability and inter-molecular interactions (reviewed in [22]). Phosphorylation of TCTP like-wise appears to be the mechanism regulating its activity, subcellular translocation, and interactions that enable it to be a player in multiple cellular processes. We propose that TCTP phosphorylation by insulin may be the stimulus that induces TCTP to translocate from membrane to cytosol and the contributory mechanism for sodium pump regulation. In this regard, our further studies are aimed to test whether insulin treatment lowers the high blood pressure of TCTP-TG mice.

Conceivably other posttranslational modifications of TCTP, such as oligomerization and truncation, may also contribute to the regulation of TCTP. Additionally, specific pathological conditions may provoke some of the posttranslational modifications and contribute to TCTP-related diseases such as diabetes, and allergy [23], and states such as chemoresistance [24]. The defined relationship between insulin dysfunctions and the role of TCTP in disease status may help to decipher the pathophysiology of diabetic hypertension. Thus phosphorylation and inhibition of TCTP by insulin may serve as potential targets for therapeutic interventions to mitigate TCTP-related diseases and conditions that might include vascular hyperreactivity, sodium pump dysregulation, hypertension, and diabetes mellitus.

## 4. Experimental Section

### 4.1. Cell Culture and Treatment

Cells from a human embryonic kidney cell line, 293T, obtained from American Tissue Culture Collection (ATCC), were cultured in cell culture dishes containing Dulbecco’s Modified Eagle’s Medium (DMEM, Gibco, Grand Island, NY, USA), supplemented with 10% fetal bovine serum (FBS, Gibco), and penicillin/streptomycin (Gibco) at 37 °C under humidification with 5% CO_2_. After serum starvation, the 293T cells were incubated in media containing 100 nM insulin (Sigma-Aldrich, St Louis, MO, USA) for indicated times. For transient expression of TCTP, pEGFP-N1 constructs containing the TCTP gene were transfected in 293T cells using WelFect EX Plus reagent (WelGene, Seoul, Korea) according to the manufacturer’s guidelines.

### 4.2. Generation of Ser to Ala Mutants of TCTP

Full-length rat TCTP was subcloned into pEGFP-N1 vector using the following primers (forward: 5'-CCC AAG CTT (Hind III) ATC ATC ATC TAC CGG GAC-3'; reverse: 5'-ACG CGT CGA C (Sal I) GA CAT TTT TCC ATC TCT AA-3'). Serine to alanine (Ser-Ala) point mutations at specific serine residues (9, 15, 37, 53, and 98, of rat TCTP), were accomplished employing polymerase chain reaction (PCR)-based site-directed mutagenesis employing Quickchange II kit (Stratagene, La Jolla, CA, USA). All point mutation constructs including pEGFP-N1-S9A, -S15A, -S37A, S53A, -S9AS15A, and -S46A64A98A (triple mutant, TM) were prepared using primers that were specifically designed to generate Ser to Ala substitution (S9A forward: 5'-GG GAC CTC ATC GCC CAT GAC GAG C-3', S9A reverse: 5'-G CTC GTC ATG GGC GAT GAG GTC CC-3'; S15A forward: 5'-GAC GAG CTG TTC GCC GAC ATC TAC AAG-3', S15A reverse: 5'-CTT GTA GAT GTC GGC GAA CAG CTC GTC-3'; S37A forward: 5'-GGC AAG ATG GTC GCT AGA ACA GAG GG-3', S37A reverse: 5'-CC CTC TGT TCT AGC GAC CAT CTT GCC-3'; S53A forward: 5'-GT GGA AAT GCT GCC GCT GAA GGT C-3', S53A reverse: 5'-G ACC TTC AGC GGC AGC ATT TCC AC-3'; S46A forward: 5'-G ATC GAT GAT GCA CTC ATT GGT GGA AAT-3', S46A reverse: 5'-ATT TCC ACC AAT GAG TGC ATC ATC GAT C-3'; S64A forward: 5'-GAA GGT ACC GAA GCC ACA GTA GTC ACC G-3', S64A reverse: 5'-C GGT GAC TAC TGT GGC TTC GGT ACC TTC-3'; S98A forward: 5'-C AAA GAC TAC ATG AAA GCA CTC AAG GG-3', S98A reverse: 5'-CC CTT GAG TGC TTT CAT GTA GTC TTT G-3'). Additional point mutations at indicated residues were accomplished as described. The point mutations at specific residues were verified by nucleotide sequencing (data not shown).

### 4.3. Western Blotting

Cells were washed with phosphate-buffered saline (PBS), and harvested by scraping from the culture dish. They were lysed with a cold lysis buffer (20 mM Tris, pH 7.5, 135 mM NaCl, protease inhibitor, phosphatase inhibitor cocktail (Sigma), 1% Triton X-100), briefly sonicated and centrifuged at 12,000 rpm for 10 min at 4 °C. The protein content of the lysates was assessed by Bradford assay (Bio-Rad Laboratories, Hercules, CA, USA). The lysate samples were diluted with sample buffer and boiled. Aliquots of the lysates containing equivalent quantity of proteins were resolved by sodium dodecyl sulfate-polyacrylamide gel electrophoresis (SDS-PAGE). Proteins on gel were transferred to nitrocellulose (NC, Whatman, GE Healthcare Life Sciences, Piscataway, NJ, USA) or polyvinylidene difluoride (PVDF, Amersham Pharmacia, Piscataway, NJ, USA) membrane and blocked. The proteins were then probed with primary antibodies against anti-β-actin (Cell Signaling Technology, Beverly, MA, USA), -GFP (SantaCruz Biotechnology Inc., Santa Cruz, CA, USA), and -TCTP (Lab Frontier, Seoul, Korea) and then incubated with a horseradish peroxidase (HRP)-conjugated secondary antibody (Bio-Rad). Protein bands were analyzed using an enhanced chemiluminescence (ECL, Amersham) and UV Products Image Reader (LAS 3000, Fuji Film, Tokyo, Japan).

### 4.4. Preparation of Membrane and Cytosol Fractions

After transfection of pEGFP N1-TCTP-GFP or its Ser to Ala point mutants, the cells were serum starved. The quiescent 293T cells were then treated with insulin (Sigma) at a concentration of 100 nM for indicated times, washed, and lysed in a buffer devoid of detergent (200 mM Tris (pH 7.5) 135 mM NaCl, phosphatase inhibitor I and II, and protease inhibitor (Sigma)) on ice. The lysates were subjected to brief sonication and centrifuged at 3120 rpm for 15 min at 4 °C. The supernatants were collected and centrifuged again at 14,000 rpm for 90 min at 4 °C. The resulting pellet gave crude membrane and the supernatant, cytosolic fractions. The pellet was then diluted in the lysis buffer now containing a detergent, 1% Triton X-100. The cytosolic and membrane fractions were subjected to SDS-PAGE and the protein bands were analyzed by Western blotting.

### 4.5. Detection of Phosphorylated Proteins in Cells

After transfection with pEGFP N1-TCTP-GFP or Ser to Ala mutant constructs, the 293 T cells were serum starved, treated with insulin (100 nM) for indicated times and their cytosolic and membrane fractions were prepared as previously described. Phosphorylated TCTP was detected by immunoprecipitation with anti-phospho-serine (p-Ser), -phospho-tyrosine (p-Tyr), and -phospho-threonine (p-Thr) antibodies (Sigma) for 1 h. The reaction mixtures were then reacted with protein A-agarose overnight in a rocking platform at 4 °C. In addition, the fractions were treated with anti-p-Ser agarose (Sigma) to immunoprecipitate the TCTP phosphorylated at its Ser residue(s). The immune complexes were washed, diluted with sample buffer containing SDS and separated by SDS-PAGE. The resolved proteins were electro-transferred to NC membrane (Whatman) and immunobloted using anti-GFP antibody (SantaCruz).

### 4.6. Prediction of Phosphorylation Sites in TCTP

To predict the phosphorylation residues of TCTP, several database servers providing phosphorylation prediction functions including NetPhos [25] and PHOSIDA [26] were used in accordance with the suppliers’ guide.

## 5. Conclusions

In the present study, we found that insulin induces phosphorylation of TCTP in 293T cells at Ser residues. Phosphorylation of TCTP by insulin was independent on the origin of substrate, endogenous or exogenous origins of TCTP, but dependent on the types of cells. Insulin specifically phosphorylates the Ser-9 and -15 residues of TCTP, possibly through the stimulation of the still unidentified insulin-dependent kinase. Phosphorylated TCTP was not found in the plasma membrane but detected in the cytosolic fraction of cells. Study on the responsible kinase and the possible involvement of TCTP phosphorylation in sodium pump regulation is needed for further verification. Use of insulin can be considered in the treatment of TCTP-induced hyperreactive vascular disease such as hypertension. 
